# Paving the way: Urban Health, Food Systems, and the Imperative for Holistic City-Led Action

**DOI:** 10.12688/f1000research.163042.1

**Published:** 2025-05-22

**Authors:** Katrina Lundberg, Ana Moragues-Faus, Lukar Thornton, Nevin Cohen, Lucy Diekmann, Luz Maria De Regil

**Affiliations:** 1 Consultant to the World Health Organization, Geneva, Switzerland; 2 Food Action and Research Observatory (FARO), Universitat de Barcelona, Barcelona, Spain; 3 Department of Marketing, Faculty of Business and Economics, University of Antwerp, Antwerp, Belgium; 4 City University of New York, New York City, USA; 5 University of California Agriculture and Natural Resources, Davis, California, USA; 6 World Health Organization, 20 Avenue Appia, Geneva, 1211, Switzerland

**Keywords:** Food systems, nutrition, food security, food safety, urban health, cities, public health

## Abstract

Most of the food produced globally is consumed in urban centres, and urban populations rely heavily on purchasing the foods they consume in place of growing, catching, or producing food themselves. Urban populations are thus highly dependent on the types of foods available to them and their relative prices. Rapid urbanization is catalysing changes across the food system and in turn, shaping urban diets. Traditionally viewed as a problem predominantly faced by rural areas, food insecurity and malnutrition (both undernutrition and overweight and obesity) are on the rise in urban areas globally. Likewise, urban environments can foster unsafe food environments, increasing the risk of foodborne illnesses and death. Considering an increasingly urbanized world, this paper describes critical links between food systems and urban health. It identifies housing development, digitalization, labour transitions and dietary transitions as emerging and interrelated trends that are changing the way people access, purchase and consume food in urban areas. This paper proposes potential interventions and tools for holistic, city-led policies and actions that make healthier and safer food more available and affordable for everyone, to inspire and encourage practitioners and policymakers at the city, regional, national and international levels to increase their ambition and act urgently.

## Key messages

1. Urban environments are spaces in which many food systems issues combine to impact health, socio-economic, and environmental outcomes.
2. Since urban populations mostly source food through purchasing, the food that is available and its relative price within the food environment is a key determinant of nutrition and health.
3. Actions to ensure urban food systems better promote health need to be informed by an awareness of contemporary social, technological, and economic trends such as those relating to, housing development, digitalization, labour transitions and dietary transitions.
4. The manifestations and outcomes of these trends are context-specific and point to the need for tailored solutions.
5. City leadership faces several challenges to developing and implementing policies for better urban health, including power imbalances and aligning action across sectors and levels of government.
6. There is a need to address urban health policy in a holistic and integrated manner while anticipating potential trade-offs of new policies.
7. Governments at every level need to consider how to ensure equitable access to healthy, safe, and sustainable foods in cities, a task that is becoming more challenging with growing urban populations.
8. City governments can capitalize on their proximity to policy beneficiaries by targeting policies and interventions to address the root causes of dietary health inequities.
9. As cities are major markets for food, urban food policies can drive beneficial changes to the entire food system.

## Introduction

More than half of the world’s population lives in urban areas, with projections indicating this figure could reach 70% by 2050, largely due to urbanization in developing countries in Africa and Asia. ^1^ Health inequities are hallmark characteristics of urban areas with populations disparately exposed to health risks including poor sanitation, unsafe foods and unhealthy diets, occupational hazards, and climatic shocks. Urban areas are also often sources of social determinants of poor health with inequalities driven by individual and household-level factors such as poverty, income inequality, race, ethnicity, gender inequalities and spatial segregation exacerbating environmental exposures. Socioeconomic and structural changes in the global food system, in part due to rapid urbanization, are reshaping the way people eat and live, with important implications for nutrition and health. Actions in urban areas led by local governments can contribute to the realization of several Sustainable Development Goals as well as ensure the attainment of the Right to Food [1].

Food systems are partially shaped by policies which determine what food is made available, to whom and at what price. ^2^ Currently, up to 70% of food produced globally is consumed by urban areas ^3^ yet many have food systems that result in unequal access to healthy, safe, sustainable, culturally appropriate, and affordable food, affecting population health. Food environments have experienced rapid changes over the last decade. ^4^ While space to grow foods in urban areas is limited, urban food environments now incorporate a broad range of food options across an array of distribution channels (e.g., street food vendors, supermarkets, traditional markets, food pantries and soup kitchens, schools and other public canteens, restaurants, fast-food outlets, meal delivery services), and various forms of food marketing, including promotion, from traditional to digital marketing.

This article is part of a series of papers that explore urban health through the lens of various multi-sectoral issues. We focus on trends shaping the interactions between urbanization, food, and health, and highlight critical interventions to inspire and encourage practitioners and policymakers at all scales to increase their ambition and act urgently to ensure that food systems not only feed urban populations but also *nourish* them.

## Findings: Key connections and food systems trends shaping urban health over the coming decade

### Food systems and urban health key connections

Food systems have direct and indirect effects on health. ^5^ They can directly influence health by determining whether people have enough food to eat (sufficiency) at all times (stability) [i.e. food security], whether people have financial access to nutritious foods, the relative availability and “attractiveness” of foods [i.e. to achieve healthy diets], and whether food is free from health-harming biological or chemical contaminants [i.e. food safety]. Food systems also indirectly influence health through their ecological, economic and social impacts.

Traditionally viewed as a problem predominantly faced by rural areas, food insecurity [2] is growing in urban areas, in part driven by rapid urbanization. Recent analysis reveals that, globally, food insecurity is only slightly lower in urban areas than in peri-urban or rural areas (25.5% vs. 29.9% and 31.9%, respectively). The greatest levels of urban food insecurity lie in Africa and Latin America (54.3% and 32.2%, respectively), while this is 11.8% in Northern America and 8.1% in Europe. ^6^ While the relative rate of food insecurity is lower in urban than rural areas, given the large size of urban populations, the vast majority of food insecure people worldwide (76%) live in urban and peri-urban areas. ^7^ However, variations exist between- and within-countries and also within-cities. ^6^ For example, food insecurity prevalence is likley to be higher in urban areas with informal settlements and migrant populations. Similarly, levels of food insecurity may be higher among women than men, though this relationship may be mediated by other factors such as education level and household income. ^8^

Urban food environments are unique in that the urban population purchases most of the foods that it consumes making urban populations highly dependent on the foods that are available to them and their relative prices. In addition, higher costs of living (and smaller housing units) which often characterise urban living, constrict household food budgets leading many households to compromise on the dietary quality of food purchases, especially as nutritious foods such as vegetables, fruits and animal sourced foods are often more expensive than highly processed foods. ^8, 9^ Other factors more prominent in urban settings, such as limited food preparation time relative to effort, space constraints for food storage and preparation, pervasive food marketing across various media, and easy physical access to foods high in unhealthy fats, sugars and/or sodium, contribute to shaping today’s urban diets. ^7^

Food insecurity, combined with these other social and environmental structures in urban areas has contributed to the rise in diet-related diseases and the persistence of the multiple forms of malnutrition we see today. Malnutrition in all its forms (including both undernutrition and overweight and obesity) is responsible for an estimated 26% of global preventable mortality. ^10^ Levels of undernutrition (stunting and wasting [3]) remain high in urban areas in Africa and Asia, affecting about 25% of children in these regions. These carry important implications for health, particularly relating to physical growth and cognitive development and increased susceptibility to, and risk of dying from, common infections. Stunting may also increase the risk of developing noncommunicable diseases (NCDs) in adulthood. In contrast, child overweight remains higher in urban areas than in rural areas, ranging from around 5% in Africa and Asia, up to 9% in Latin America and the Caribbean. Overweight and obesity are important risk factors for several NCDs including diabetes, heart disease and certain kinds of cancer. ^8^ However, these profiles are changing around the world as overweight and obesity levels continue to rise, while levels of undernutrition remain stagnant. ^6^ The relative rates of these transitions in malnutrition profiles across settings can have important implications for the burdens that national and local governments face. Several countries and urban settings now face the co-existence of multiple forms of malnutrition, known as the ‘double burden of malnutrition’. ^6, 8^

Beyond the healthfulness of diets, the safety of the food supply in urban areas is equally important since even if nutritious food is available, it cannot benefit individuals if it is unsafe. Food safety issues may arise across the food system from production to consumption. Within urban settings, inadequate or poorly enforced regulatory standards together with densely populated urban environments can foster unsafe food environments, including from unsafe water, poor food production and handling practices, and inadequate food storage infrastructure ^8^ [See Box 1 for more details on specific urban settings]. Even where fresh foods sold through traditional markets or supermarkets are safe, food safety issues can be introduced in home meal preparation, especially where safe water and adequate storage or refrigeration are not guaranteed. Food contaminants have been linked to a wide range of health outcomes, from acute gastrointestinal illnesses, to poisoning, to long-term development of certain cancers. ^11^

Box 1. **Critical contexts for urban food safety: Traditional markets and street food vendors.**

In many urban settings, markets represent an important source of fresh fruits, vegetables, and other perishable foods, especially for low- and middle-income groups (though in high-income countries and some areas of low-income countries gentrification and ‘touristification’ of traditional markets have had negative consequence for local and lower-income consumers and market vendors affecting the types of foods sold and pushing up prices). ^7^ However, these foods also increase the risk of foodborne diseases when a lack of hygiene prevails. Traditional markets may also present risks with respect to emerging zoonotic diseases. Another hallmark of urban food environments, street foods, represent an important source of daily meals for an estimated 2.5 billion people globally, especially in instances where housing is tight and densely populated, and for low-income workers. ^8, 12^ However, infrastructural and regulatory gaps along the street food supply chain – characterized by informal street food vendors who may have only temporary structures without running water, cold storage, and sanitation facilities- increase the risk of food-borne diseases. These issues could reinforce unhealthy diets, as more easily produced, often fried, and low-cost street foods or highly processed foods may be consumed instead. In addition, a lack of public trust in the safety of local foods or products may shift consumer purchasing towards imported products, or more processed products with longer shelf-lives but also often worse nutritional profiles. ^8, 13^

While new estimates are set to be published in 2025, the latest available data from 2010 suggests that globally, foodborne hazards may cause over 600 million cases of illness and 420,000 deaths annually. ^14^ Children are disproportionately affected by foodborne diseases, with 40% of cases occurring in children under age 5 years. Likewise, burdens are not distributed equally across the globe. The African region faces by far the greatest burden of food-borne diseases followed by the South-East Asia and Eastern Mediterranean regions. ^14^ As cities experience the impacts of climate change including extreme weather events (flooding) and heat islands, their food supply systems become increasingly vulnerable to food safety concerns with concomitant implications for food security and nutrition. ^7^

Ultimately, urban food systems have a significant impact on health, with food insecurity and malnutrition remaining critical issues for cities worldwide. The unique challenges of urban environments demand tailored context-specific solutions to improve health outcomes for urban populations. As urban populations grow, it becomes increasingly important to understand the underlying factors that help shape urban food systems and their impact on health, to develop effective strategies for addressing these pressing issues.

## Trends shaping urban food security and nutrition

**Figure 1.**
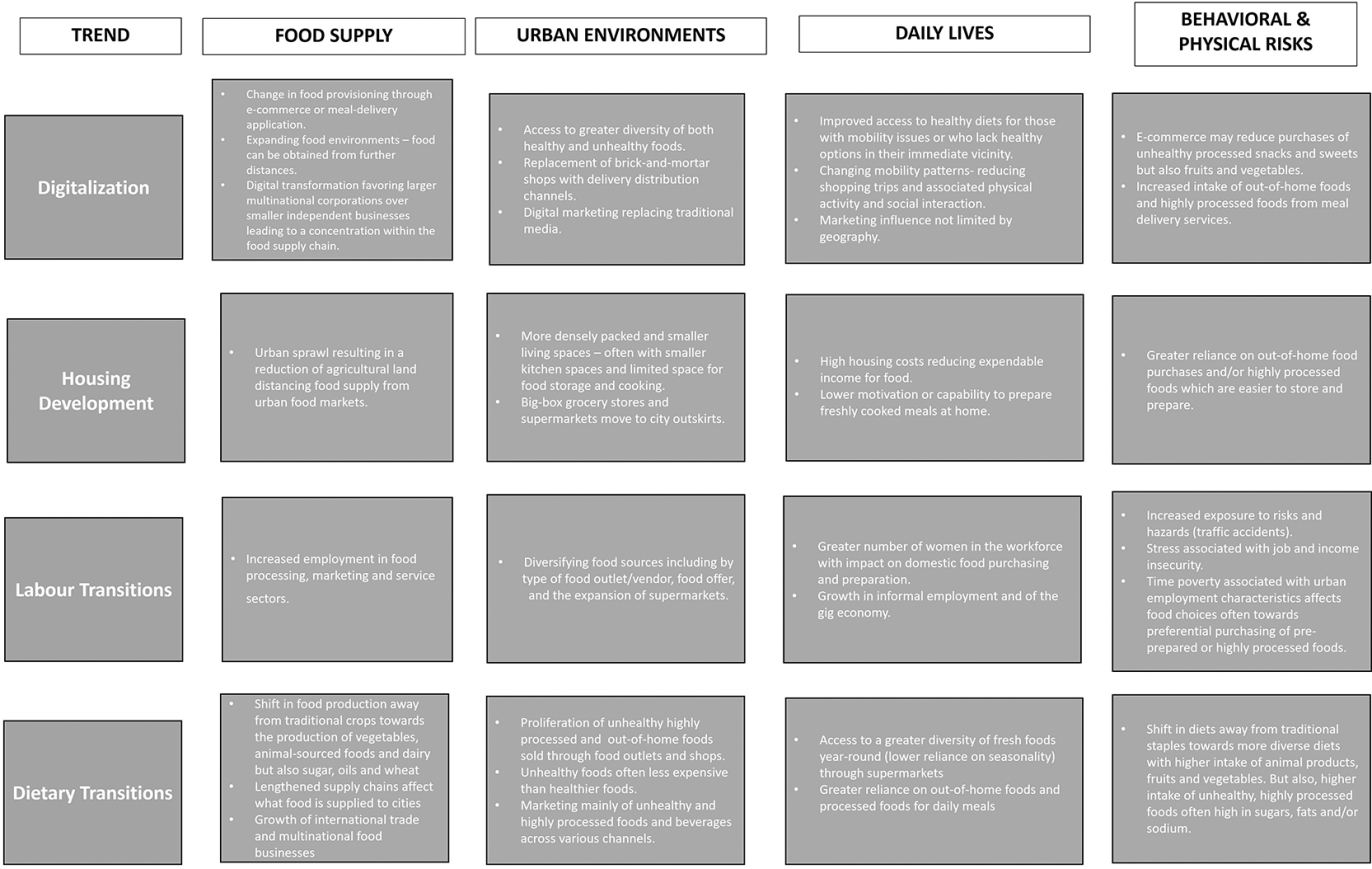
Sample impacts of food systems trends across the urban food system.

While there are different studies exploring the diverse environmental, technological, economic, political, sociocultural, and demographic drivers shaping urban food systems and associated health outcomes for urban populations explored above. ^8, 15^ In this paper we address four emerging and interrelated trends that to date have not been well covered in the literature, and describe how they are changing urban food systems and urban health, namely: 1) housing development; 2) digitalization; 3) labour transitions; and 4) dietary transitions (outlined in Figure 1).

### Housing development

Housing remains a critically undervalued element in the discourse on healthy urban food systems. Whilst housing issues such as crowded housing and poor cooking facilities were previously acknowledged in a WHO report, ^16^ this section focuses on three ongoing aspects of housing that pose significant challenges to food security and nutrition: housing design, urban planning (“urban sprawl”), and economic policy (“cost of living”).

Firstly, the ongoing pressure to accommodate increasing populations in urban areas has led to a proliferation of smaller living spaces. There is an inherent tension between the commonly cited benefits of urban density and building upward (higher wages, green space preservation, decreased energy consumption, walkability, and access to services), and other measures of quality of living spaces (including size and utility). ^17^ While this trend is not inherently negative due to broader urban benefits brought about by compact living design, at the housing level it often results in diminished kitchen sizes and functionality. Consequently, if cities place increasing emphasis on the provision of affordable nutritious foods by local retailers, this may yield limited advantages if residential accommodations make refrigeration and at-home meal preparation increasingly difficult. Furthermore, the provision of inadequate storage space impedes residents’ ability to purchase food in bulk, a cost-effective strategy commonly employed to manage food budgets. ^18^ Additionally, as housing influences the types of food we can store and prepare, this shift in food practices bears implications for food supply chains, which are responsive to consumer demands. The recent surge in meal delivery systems (described in greater detail below), propelled by the COVID-19 pandemic but also reflective of urban lifestyles and housing models, characterizes this trend. In response, recent housing design standards (e.g., in the Greater London area), have incorporated detailed guidelines specifically targeting kitchen design, aimed at bolstering size and functionality. ^19^

The second concern related to housing pertains to urban planning (“urban sprawl”). Both push and pull factors contribute to the expansion of residential land far from city centres. This growth has been shown to manifest differently in different contexts. In high-income countries, growth in city outskirts is typically considered a positive measure of urban welfare, characterized by suburban or low-density development. Properties in these areas usually cost less, there is more space for larger homes and gardens, environments are generally quieter and more natural, and there are social (and sometimes agricultural) opportunities, leading some to favour such areas. However, this lower-density setting also impacts the food retail landscape, with larger chain and big-box retailers taking advantage of lower commercial land costs and expanding road transport infrastructure often displacing smaller, independent shops. The placement of these retailers, typically segregated from residential areas, exacerbates car dependency with negative environmental and health consequences.

Further, vulnerable and disadvantaged groups, including migrants, may be relegated to lower-cost, lower-quality areas further from economic and cultural opportunities. In low-middle-income countries, this growth is often characterized by informal or illegal, unplanned urban expansion, such as shanty towns in Africa and slums in Asia [see Box 2 for more detail on informal settlements and slums]. ^7^ In both cases, such expansion often encroaches upon land that is, or could be, utilised for agricultural purposes, thus distancing the food supply from urban cores. Despite ongoing dialogue on optimal urban design, the construction of new, inadequately planned urban areas on the peripheries of major cities persists.

Box 2. **Food security and nutrition in slums and informal settlements.**

Globally, one billion people (representing 25% of people in urban settings) live in informal urban settlements (slums), and this figure is expected to double within the next 30 years. Food insecurity is relatively high in informal and slum settings largely because of poverty and poor living conditions. Low-income housing, especially in informal settlements, often lacks key infrastructure such as kitchen and bathroom spaces and thus food may be cooked in communal areas with limited space for food storage and preparation. ^7^ In addition, access to food, particularly fresh and nutritious food, is often limited in slum settings, with open and wet markets, as well as street food vendors and informal kiosks remaining the main food source. ^8^ Together with other factors such as the cost of fuel for cooking, these conditions may influence food choices towards pre-prepared foods. The same living conditions factors may exacerbate malnutrition due to increased exposure to communicable diseases, including vector- and food-borne diseases and others. Contrary to popular belief, several studies in urban settings in low-middle-income countries have revealed the double burden of malnutrition to be present in informal settlements, with neighbourhoods and households exhibiting both surprisingly high levels of overweight and obesity and high levels of undernutrition (stunting and micronutrient deficiencies). ^7^

The third issue concerns the cost of housing, whether through ownership or rental. With an increasing proportion of household budgets allocated to mortgage or rent payments, pressure mounts on household food budgets. For example, recent census reports from New York City, USA indicated that housing is typically the largest expense for families with more than a third of household income dedicated towards rental payments. ^20^ Combined with rising food prices, households face immense pressure to meet their basic nutritional needs and may cut back on their food expenditures to compensate. This trend fosters the purchasing of cheap, nutrient-poor, energy-dense and undiversified foods. Though social protection and food assistance programmes aimed at improving access to a healthy diet exist in many cities, these are often insufficient to fully address food insecurity as they do not cover all household food needs or other living expenses (health, housing, education, etc.), nor do they address barriers to cooking (such as space and storage constraints). ^8^ In such instances, it is clear a single policy response is inadequate; instead, a multifaceted response targeting the multiple issues is required.

### Digitalization

Urban food environments are changing due to digitalization, with significant health and structural consequences, both positive and negative. New digital platforms that connect consumers, retailers, and workers through apps and GPS-enabled mobile devices have changed the market for home-delivered food while also creating a large, precarious, and underpaid labour force of “food gig workers.” Similarly, digital marketing of food has become ubiquitous, targeting—aggressively and with precision—the consumer segments most vulnerable to buying inexpensive, unhealthy food: high in unhealthy fats, sugars and/or sodium, and typically highly processed. ^21^ These innovations have been adopted very quickly and have diffused worldwide, affecting urban food environments before policies and programmes have had a chance to catch up to protect consumers and workers.

Some of the most critical changes caused by digitalization, particularly for urban policy, affect food provisioning. Digital food provisioning includes e-commerce food retail channels that have transformed how some consumers select and buy food. ^22^ Many of these changes have emerged in the past decade, with adoption accelerating in 2020 due to the COVID-19 pandemic as distancing precautions, business restrictions, and population lockdowns caused businesses and consumers to shift to online sales channels for food provisioning. ^22– 24^ Since then, online shopping for both groceries and prepared meals has continued to grow, and food has been forecasted to be the fastest-growing category of e-commerce sales worldwide. ^25^

Digitalization has the potential to benefit those populations with inadequate access to healthy diets by increasing the convenience and access to nutritious foods, with positive implications for health and equity. ^15^ However, with unhealthy foods aggressively promoted and readily available on digital platforms, digitalization can also limit healthier and increase the availability and “attractiveness” of unhealthy choices and lock people into potentially unhealthy buying and consumption patterns. Findings on the impact of online groceries on diets are mixed, with some studies finding healthy food consumption associated with online grocery use and others finding online shoppers less willing to buy perishable food than shelf-stable and often less healthy options. ^26^ The growth of delivery platforms has also made it easier for consumers to buy food from restaurants. However, the food offer on delivery platforms is often predominantly unhealthy. ^27^ Food safety is an additional concern, as proper handling practices across the delivery supply chain are rarely considered. ^8^ Although online purchasing can ease access for mobility-limited consumers, this positive impact may be constrained by other disparities such as the digital divide.

Digitalization can also harm “brick-and-mortar” [4] food retailers, eventually resulting in fewer (and potentially less healthy) buying options. For example, while it may increase retail competition in areas under-served by brick-and-mortar supermarkets, digitalization may also advantage global online retailers with advanced supply chains, undermining independent grocers and leading to market domination. Platform-based food delivery companies may enable small independent businesses to access new consumers, in turn affording consumers more convenient access to groceries and prepared meals. ^8^ However, for these small businesses, platform fees may cut into profits, potentially resulting in a concentration of large transnational chains that can buffer the higher costs, at the expense of smaller local family-owned businesses.

Another aspect of digitalization is linked to the provision of food information through technology, and the extent of information necessary for, and to be retained on, physical labels and information made available when purchasing foods online, and through meal delivery apps.

### Labour transitions, particularly in the food sector

Across low-, middle-, and high-income countries, the food system is a major source of jobs and economic activity in urban areas. ^28, 29^ Food system workers are employed in different sectors of the food value chain, where they produce, process, transport, and sell food as well as manage food waste. Overall, food system business and employment make up a large part of urban economies, while cities’ food service and retail sectors—e.g., restaurants, cafes, food vendors, and markets—contribute to food access and the unique character of urban areas, which includes the culture and variety of cafes and restaurants among others. ^29^ Increased demand from urban consumers for nutrient-rich foods as well as processed and convenience food is a driver of job creation along the food value chain, particularly in the processing, marketing, and service sectors. ^8^ Additionally, recent decades have seen a rise in female employment outside the home; a recent study in west Africa found that women made up the majority (80%) of those working in food processing and vending sectors. ^30^

Given these trends, growth in food system employment and food system enterprises has the potential to provide economic opportunity and jobs for expanding youth and other vulnerable populations. ^8, 28, 31^ However, food system jobs are also widely critiqued for their low wages, job insecurity, lack of adequate health benefits, difficult or dangerous working conditions, lack of opportunity for advancement, and precariousness. ^32, 33^ Informal food sector activities and casual labour are particularly important to low-income urban populations, though they provide only low daily earnings. ^7^ The rise in demand for digital services (including food provisioning) is accompanied by a growing demand for online gig work. Today the ‘gig economy’ [5] is estimated to account for up to 12 percent of the global labour market and, while still dominated by high-income countries, demand in low-middle income countries is increasing and at a faster rate. ^7^ Digital platforms have recently come under scrutiny for the way that they treat gig workers as independent contractors, not employees, meaning that these workers do not get the protections and benefits of conventional workers, including compensation for workplace injuries, sick leave, and collective bargaining rights, among others. Overall, non-standard or unstable jobs have been documented to be more common among lower-grade occupations, women, ethnic minorities and immigrants. ^34– 36^

Poor working conditions and low wages have direct and indirect consequences for health, including lack of access to health care and inadequate housing. The type and conditions of urban employment are also central to ensuring urban food security because urban residents primarily obtain food by purchasing it with the income they have earned through work. Those living in poverty—including low-wage food system workers—are at increased risk for food insecurity and may consume energy-dense foods of low nutritional quality because that is what is most affordable. ^8, 37^ The COVID-19 pandemic and associated lockdowns exemplify these vulnerabilities for urban workers. Food retail, food service, and hospitality industries were disproportionately affected by measures to reduce the spread of disease, as many workers were either considered essential to the maintenance of critical infrastructures and thus risked greater exposure to infection, or faced job insecurity due to business and border closures (for seasonal migrant workers). ^38^

In addition to the negative health implications for food system workers, these food labour practices pose food safety risks for the wider urban population when workers handle food while sick, which is particularly important to consider in countries without social healthcare, as workers are less likely to have workplace-provided health insurance. ^37^ Physical risk factors such as stress, environmental impacts, occupational hazards, and low wages, have been associated with poor physical and mental health outcomes. ^39^ These can have a double impact when they affect the main household earner potentially simultaneously reducing access to nutritious foods for entire families. ^8, 35^

Changes in employment including increased out-of-home female employment can have implications for food purchasing and consumption patterns in the global North and increasingly in the global South. Since women often still carry the primary responsibility within the household to purchase and prepare food, additional time spent out of home and commuting may drive increased purchasing of pre-prepared convenience and highly processed foods which are often less healthy than meals prepared at home. Several studies across contexts have found positive associations between women’s empowerment and household dietary diversity. ^6^ However, as this diversity may also include an increased selection of unhealthy foods high in fats, sugars and/or sodium ^40^ complementary interventions which target urban food environments will also be key.

### Dietary transition

Another interconnected trend over the past decade affecting human health and affected by food systems is dietary transitions, characterized by a dramatic shift in the types and quantities of foods and beverages consumed, towards more diverse diets (higher in animal sourced foods, fresh vegetables and fruits) but also greater consumption of unhealthy, highly processed foods high in unhealthy fats, sugars and/or sodium. ^8^ Consequently, increased dietary diversity has not automatically resulted in improved diet quality, with significant differences across regions and socioeconomic status in urban areas. Key drivers of this trend include globalization of food systems, urbanization (and the points discussed above: housing, digitalization and labour participation, especially of women), economic growth and income growth. ^41, 42^

Many factors associated with dietary transitions converge in urban areas, making them particularly vulnerable to their impacts on population health. Urban food systems are commonly characterized by more diverse food sources (food vendors, restaurants and supermarkets), high exposure to food marketing, and limited opportunities to grow nutritious foods. The result is greater access to and demand for a broader range of food options. One major shift, especially in Asia and Latin America, has been the expansion of supermarkets and hypermarkets at the expense of conventional markets, largely because of policy liberalization and privatisation, public infrastructure investments and globalized distribution of modern food technologies, and demand for readily available and safe foods. Today, supermarkets are often part of multinational chains or dominant national chains. While supermarkets may increase access to safe and nutritious food, year-round, they have also been associated with the increased supply of unhealthy processed foods. Supermarkets may also displace local food businesses (both formal and informal) with important ramifications for food security and market consolidation. ^7, 8^

Within urban areas, neighbourhood food environments differ significantly, especially with respect to opportunities to access healthy diets, as mentioned above. Some urban areas have no or only few food shops or outlets that sell fresh or healthier foods (food deserts), while others have a high density of shops and outlets that serve mainly energy-dense or highly processed foods high in unhealthy fats, sugars and/or sodium (food swamps). Often these are found in under-invested neighbourhoods, for example in Rio De Janeiro, Brazil, in which residents of low-income communities (such as those in impoverished or informal neighbourhoods) and low-income households living in affluent areas, often have limited access to healthy diets. ^43^ The availability and the affordability of a healthy diet is especially significant as urban populations rely heavily on food purchases. ^7^ The fact that the relative price per calorie is often lower for unhealthy foods than for healthier foods, across regions and country income groups, also has important implications for diets. For example, in Hanoi, Viet Nam lower-income groups’ food purchases account for 46% of total household expenditure. ^8, 35, 42^ An analysis of food consumption in deprived neighbourhoods of three cities in Ghana and Kenya revealed widespread consumption of unhealthy (mainly local) foods, often in lower socioeconomic status groups. Similar findings have been documented in high-income countries. ^44^ Opportunity of time and convenience of meal preparation are important co-factors governing food choices and diets in urban settings (explored above).

Urban growth and dietary changes also affect food production and supply. Growing demand for certain products, including specific crops, vegetables, animal-sourced foods, and dairy products can have important effects on food production and supply chains, shifting production away from traditional crops and lengthening supply chains. For example, according to some studies, a greater demand for vegetables and dairy products in New Delhi, India, in both high-, low- and middle-income urban households, has resulted in a shift in production in the rural areas around New Delhi away from cereals towards vegetable production and livestock keeping. ^45^ Expansion in the production of cheap raw ingredients such as sugar, oils, and wheat as well as food processing technologies is also feeding the growth of highly processed food industries and changing diets globally, though at different rates. ^6^ In addition, increased demand for foods and products with a high environmental footprint, such as pre-packaged highly processed foods, or animal-source foods requiring significant resources (including water, energy, and land), may hurt the environment and exacerbate climate change, ^46^ including through increased emissions. ^47^

The four trends described above are interconnected (see Figure 1). Housing design may discourage home cooking, leading residents to rely instead on out-of-home food purchases from street food vendors or meal delivery services. Digitalization while making meal delivery options more convenient, can also increase precarious employment conditions which in turn will have an impact on housing and food affordability. Dietary transitions facilitate the rise in the production, distribution and sale of unhealthy highly processed and convenience foods, re-enforcing changes in urban environments related to food delivery services and home food preparation. In light of these trends, certain key issues need to be considered by national and local leadership to raise the ambition and effectiveness of urban food policies. The following section delves into the shared challenges behind the trends discussed above and uncovers opportunities to reverse their harmful health impacts. By seizing these opportunities, cities can become powerful catalysts for positive change, driving a healthier, more sustainable food system for all.

## Key challenges and potential opportunities for urban food systems policies

There are multiple potential implications on urban policy and actions that are critical to improve urban food systems. First, to address these trends we must acknowledge the role of **power at the intersection of urban areas, food and health dynamics**. Since 70% of all food produced goes to urban centres, ^3^ changes in food consumption in urban areas are an important driver to the production of more nutritious and sustainable foods that benefit broader populations. ^8^ Urban areas, particularly cities, are critical sites for addressing global sustainability and health challenges. They are essential to transforming current food systems, developing innovations, and enacting changes to ensure a liveable planet to address future crises. What action will be taken depends on the governance of cities which differ in powers and capacities across the globe and are shaped by actors operating at multiple scales. For example, city governments in Latin America are relatively strong compared to those in Africa, where decisions on urban issues are at the ministerial level. ^7^ Not only do competencies and levels of decentralisation vary significantly across the globe, but also capacities to act are spread across types of actors and scales. For instance, resources within the public sector to make investments or develop programmes can be held at different administrative levels. Examples range from where powers lie to regulate labour conditions, reform school meals or establish social programmes to guarantee access to adequate food, but also how sometimes city councils decide to dedicate additional budgets to increase social transfers, build social housing or lobby higher government levels for change such as intervening in rent prices. Civil society also plays a significant role in shaping urban food systems by developing community initiatives and shaping policy-making processes. ^48^

Private economic interests at all levels have a great power to shape urban food systems dynamics. A clear example is the expansion of international food retailers and the ‘supermarketisation’ of urban food supply which is increasingly concentrating the control of the food chain in a few powerful corporations. ^49^ Investment funds and housing companies are also shaping cities and their food systems at large, by contributing to processes of gentrification and ‘touristification’ involving increases in housing prices, displacement of the local population and transformation of food retail environments. ^50^ Power struggles present challenges to public health as powerful corporations (such as transnational food and beverage corporations) use their economic power to prevent or favorably shape political action to improve food systems and diets (through lobbying and other means). ^37^ Adopting an integrated, transparent, and participatory approach to policymaking—one that actively involves civil society and those most affected while preventing conflicts of interest—is essential to addressing these challenges.

This diversity of powers has been increasingly mobilised through different forms of multistakeholder participation, including the establishment of partnerships and participative urban policy-making processes or governance spaces such as food policy councils. These spaces have contributed to adopting a multi-actor, multi-sector, and integrated approach to urban food reform but have also surfaced power inequalities, competing values and interests, ranging from ensuring the right to food to delivering economic benefits. These unequal power dynamics lie at the root of many health inequities and have resulted in a call for a more transparent and critical undertaking of how power asymmetries play out in multi-actor spaces and partnerships as well as how they influence the development and outcomes of specific policies and interventions. Ensuring that members of multistakeholder mechanisms align on shared principles or goals, regularly review their practices to maintain consistency with these values, and remain aware of potential power imbalances will be key to mitigating such risks. ^7^ It will be vital for city governments to safeguard against conflicts of interest in all policy making and implementation by adopting a clear framework to avoid them and ensuring impartiality, accountability, and transparency in policymaking.

The second aspect relates **to a clear need for policy coherence across scales and sectors as well as for adopting a place-sensitive and strategic approach to designing and implementing interventions.** The adoption of complex and systems thinking has showcased the interdependencies between urban, food and health systems components and, therefore, potential interventions. However, these relations are shaped by multiscalar dynamics and manifest differently in each place. ^51^ A growing number of urban food policies across the globe adopt an integrated food system perspective (for example, the increasing number of signatories of the Milan Urban Food Policy Pact [6] and the WHO Partnership for Healthy Cities [7]). The challenge for these comprehensive strategies is to move beyond harvesting the low-hanging fruit towards translating plans into real high-impact investments according to their specific urban context. The allocation of resources to activities that can deliver multiple benefits has been determined to be critical as well as the mobilisation of actors operating in different scales and areas. In addition, cities often do not have direct jurisdiction over food production or over certain nutrition policies such as food labelling (which usually falls to rural areas and under national or state governments respectively). For that purpose, it is essential to coordinate policies and interventions at different scales - including urban, local, subnational, and national governments - but also to align the strategies of different actors across sectors to avoid wasteful use of time and resources. One clear example involves repurposing the multi-billion-dollar agricultural supports that harm the environment and human health. At the urban level, this could mean working to ensure access to nutritious food in publicly funded settings by connecting local agriculture with public food procurement and service plans that support city-run hospitals, departments, and other public institutions. ^9^

Finally, the current functioning of urban, food, and health systems is resulting in winners and losers on multiple dimensions, including health and wellbeing, economics, inequality, and environmental impacts. **Enacting transitions will inevitably have trade-offs that can and must be anticipated and minimized**. For example, introducing or scaling food inspection systems can contribute to improved food safety and food security as well as enhanced consumer awareness and trust. However, in many settings such inspection services remain cumbersome, costly and/or lack transparency. Compliance with new rules may result in higher consumer prices for such foods or in the exclusion of smaller businesses, which may in turn have important knock-on effects on incomes and health. ^13^ Fair labour practices, as enumerated by the International Labour Organization, include a living wage, health care benefits, freedom from discrimination and unequal pay, collective bargaining, worker rights to challenge employer abuses, and safe working. ^31– 33^ However, such practices are also accompanied by significant economic challenges for those who want to implement them. Nonetheless, fair treatment of food system workers is critical for food system resilience, provides an opportunity for inclusive urban community development, and promotes health. ^28, 32, 35^ Carefully planning urban food system transitions would help identify win-win solutions, trade-offs, and risks. In this regard, it is important to **undertake bold action to maximise beneficial outcomes but also to plan and put measures in place that mitigate the potential negative impacts of those transitions and support actors in making those changes**. A recent example comes from Denmark’s Plant-based Action Plan to boost plant protein production and consumption, to reduce Denmark’s environmental footprint and contribute to its public health. The plan includes greener public food agreement, changes in official dietary guidelines, support for switching to organic produce and a plant-based food grant to support funding the transition. ^52^

### How can a holistic, integrated approach to urban food systems address health and societal challenges?

**Multi-faceted policies** and initiatives aimed at addressing urban poverty—including issues such as employment, adequate housing, access to digital technology, and urban planning for food access—are important strategies for ensuring urban food security. At the city level, initiatives that support local food economies can encourage sustainable livelihoods and provide vital support to small-scale food businesses. Equally important is to protect informal and small food businesses from negative trade and regulatory impacts. These comprise both a large workforce in many urban areas and are an important supplier of food for those employed in the informal sector. Because of the connections between poverty and food insecurity, considering the needs of low-income workers in urban policy decisions can help mitigate negative impacts on their access to food and consequently their health. ^20^

**By taking a setting-based approach**, city governments can tailor policies and interventions to meet residents’ specific needs whether at home, in schools, at the workplace, or in digital environments. ^29^ This approach is contrasted with the traditional top-down policy frameworks that focus on individual sectors, treat symptoms rather than root causes, and overlook the needs, priorities, and experiences of people and communities on the ground. ^53^ In this way, cities have been pioneering a holistic approach to food systems policy using systems thinking to bring together actors from multiple sectors along the food supply chain. This collaborative and multi-faceted approach is essential for tackling the complex dynamics that drive the food system. ^51^ For example, in 2019, the Municipality of the Metropolitan District of Quito, Ecuador endorsed the Quito Agri-Food Strategy. This strategy, developed by multiple actors including government entities at various levels, advocacy groups, international actors, academia, and the private sector, enables agrifood systems to be included in city planning tools. ^8^

The interconnected nature of the challenges presented above highlights the **need to incorporate food and food security in policy making across sectors**. This ‘Food in All Policies’ approach, which mirrors the WHO’s Health in all Policies framework, ^54^ offers an integrated and systems-based approach to food policy making. By engaging a wide range of stakeholders, this approach demonstrates how food can help achieve their aims or the role that their polices play in achieving food-related goals. ^55^ In addition to adopting the ‘Food in All Policies’ approach, policy makers should embed a human rights approach in urban food policies and interventions, recognizing the intersections between the right to food, the right to the city, and the right to health. **Working horizontally across sectors and vertically across multiple scales will be crucial to addressing some of the structural barriers to food systems change**. For example, it has been two decades since planners began to call for attention to food systems, ^56^ yet the ongoing urban housing crisis and continued urban sprawl demand a renewed effort to integrate planning, housing regulation, and food systems. This will help create a better understanding of the effects of urban development and create policies that ensure homes are places in which it is possible to prepare, store, and recycle food safely. Beyond a systems approach, optimal food system policy must also prioritize equitable and inclusive policy processes that empower those most negatively affected by the food system to define its problems and shape the solutions. ^57^ Processes that promote agency within the food system and centre the voices of marginalized communities are important for the equitable transformation of the food system. ^7^

## Knowledge gaps and research needs

Several knowledge and research gaps arise from the previous considerations and need further exploration if we want to better understand and address issues of food systems and urban health. Briefly:

• First, the literature is increasingly drawing attention to the need to better understand and consider power dynamics and political economy within individual cities and across city networks and levels of governance. Particularly, mapping out which stakeholders benefit, which lose out, and who is left out of policy making will be key to realizing a truly participatory and inclusive urban food system.
• Second, a major issue for urban policymakers is the lack of data both on levels of malnutrition across urban areas and on the nutritional quality and safety of urban diets. Although urban food environments themselves are well characterized, more detailed information is needed on the diets of urban populations and more specifically of different socio-economic groups, and how they differ by gender in urban settings and across degrees of urbanicity. Such data will be key in understanding precise local challenges and needs.
• Third, food marketing is pervasive and becoming more targeted and efficient as advertisements are integrated into all forms of digital communication. We need to better understand and regulate food marketing (including by assessing whether existing policies restricting marketing are fit-for-purpose) to ensure that it does not shift consumer behaviours in ways that undermine nutrition, health, and the food system.
• Fourth, measuring the true cost of living in cities, including major costs such as housing, will be necessary to inform policies that can ensure that all residents have adequate resources to buy and eat healthy food.
• Fifth, there is limited empirical data to understand the impacts of digital food provisioning and how to ensure that the effects are beneficial to society. Programs designed before the wide availability of digital food buying may have to be redesigned or jettisoned as food environments and consumer practices change. Further research is also needed to fully understand the impacts of algorithmic management on food delivery workers (regarding the scale and scope of this growing workforce, the mental health and physical impacts, and a better understanding of the equity impacts of platform labour). Forecasting these impacts can support the development of fair and equitable policies and programmes. Unless we consider the effects of digitalization on our food systems, we risk repeating policy mistakes of the past.

## Conclusion

In this paper, we explored how the trends of housing, digitalization, labour, and dietary transitions are shaping today’s urban food systems and intensifying food insecurity, malnutrition, and urban health challenges. Urban food systems are increasingly recognized for their role in perpetuating health and social inequalities, and environmental harm. Meanwhile, urban governments are stepping up to solve these pressing issues by engaging in food policy aimed at reducing social and health inequities, and environmental challenges. ^29, 51^ City governments are increasingly aware of their vital role in ensuring all populations have adequate and sustainable access to nutritious food, recognizing the positive effect this can have on the health of the planet. City governments’ involvement in food policy has thus blossomed, with cities serving as places for experimentation and innovation, developing locally adapted solutions through participatory governance that brings together actors from different sectors of the food system to facilitate equity, health, sustainability, and resilience. ^51, 58^ The trends and challenges discussed throughout this paper point towards the need to address urban health policy holistically, anticipating potential trade-offs while focusing on long-term transformative outcomes. Cities have the power and possibility to reverse the health-harming impact of these four trends to become a source of health promotion. By addressing urban food system issues, cities will not only improve human health within urban centres but also create broader positive impacts across the entire food system, benefitting communities far beyond city limits.

## Ethics

Ethical approval and consent were not required.

## Data Availability

No data are associated with this article.

## Footnotes

^1^
The attainment of the right to food is the “right to have regular, permanent and free access, either directly or by means of financial purchases, to quantitatively and qualitatively adequate and sufficient food corresponding to the cultural traditions of the people”. *https://www.ohchr.org/Documents/Publications/FactSheet34en.pdf*

^2^
A situation that exists when individuals “lack regular access to enough safe and nutritious food for normal growth and development and an active and healthy life”. https://www.fao.org/hunger/en

^3^
Stunting is defined as low height-for-age, reflecting a past episode or episodes of sustained undernutrition (poor nutritional intake in terms of quantity and/or quality and/or poor absorption and/or poor biological use of nutrients consumed because of repeated instances of disease). Wasting is defined as Low weight-for-height, generally the result of weight loss associated with a recent period of inadequate dietary energy intake and/or disease. See: *FAO, IFAD, UNICEF, WFP, WHO. The State of Food Security and Nutrition in the World 2024 – Financing to end hunger, food insecurity and malnutrition in all its forms. Rome; 2024.*

^4^
“Brick-and-mortar” food retailers in this paper refer to traditional street-side businesses that serve customers face-to-face.

^5^
In this paper referring to short-term contracts or freelance work.

^6^

https://www.milanurbanfoodpolicypact.org/

^7^

https://www.who.int/teams/social-determinants-of-health/ambassador-programme-for-ncds-and-injuries/partnership-for-healthy-cities
