# Supplementary material for: Paving the way: Urban Health, Food Systems, and the Imperative for Holistic City-Led Action
Source: F1000Res. 2025 May 22;14:513. [Version 1] doi: 10.12688/f1000research.163042.1 (PMC12374149; doi:10.12688/f1000research.163042.1)
Supplement: Peer review history

# Reviewer report: Reviewer response for version 1

**DOI:** 10.5256/f1000research.179337.r391318

**Published:** 2025-09-05

**Authors:** Mariana Torres Arroyo

**Affiliations:** 1 Environmental Science, American University, New York, District of Columbia, USA

Thank you for the opportunity to review this manuscript. I enjoyed reading it and appreciated how the authors wove together interrelated trends–housing development, digitalization, labor transitions, and dietary transitions—to explain how these are shaping urban food systems and health.

 Positioning cities at the center, the manuscript notes the close relationship between urban food systems and the broader food system: urban populations, who rely predominantly on purchased food, face food insecurity, malnutrition, and food safety challenges, but also have also have the power to shape the food system itself. The authors emphasize that city governments, given their proximity to communities and role as major food markets, are well positioned to drive systemic change but must navigate power imbalances, fragmented governance, and trade-offs. They recommend holistic participatory approaches that integrate food into wider urban planning and policy frameworks to achieve healthier, more sustainable, and more equitable food systems.

 Overall, I found the manuscript relevant and well written. I think it offers a comprehensive systems-oriented overview that integrates social, technological, and economic dimensions, and its discussion aligns well with current debates in urban health and food policy. With minor edits, I believe this manuscript offers an interesting contribution to this debate.

**Specific Feedback:**

- Urban agriculture was only tangentially mentioned *(“While space to grow foods in urban areas is limited, urban food environments now incorporate a broad range of food options across an array of distribution channels”*) but might be an important link in the discussion.
- I would have liked to see lessons learned from policies that are at least partially working.
- I like the simplicity of Figure 1 but I think its presentation/quality could be improved.

**Minor list of improvements that could be incorporated to enhance the manuscript:**

  

- The following text could be linked to literature:
  - *“This diversity of powers has been increasingly mobilised through different forms of multistakeholder participation, including the establishment of partnerships and participative urban policy-making processes or governance spaces such as food policy councils. These spaces have contributed (…) These unequal power dynamics lie at the root of many health inequities and have resulted in a call for a more transparent and critical undertaking of how power asymmetries play out (…) as well as how they influence the development and outcomes of specific policies and interventions.”* (p.20) → Literature on policy councils is widely available.
  - *“The second concern related to housing pertains to urban planning (“urban sprawl”). Both push and pull factors contribute to the expansion of residential land far from city centres. This growth has been shown to manifest differently in different contexts. In high-income countries, growth in city outskirts is typically considered a positive measure of urban welfare, characterized by suburban or low-density development (…)”* → This is a useful discussion but would be stronger if linked to evidence. Otherwise the authors may consider rephrasing.
  - “The food offer on delivery platforms is often predominantly unhealthy.” → This claim could be more cautiously framed, unless stronger evidence can be cited.
  - “Second, a major issue for urban policymakers is the lack of data both on levels of malnutrition across urban areas and on the nutritional quality and safety of urban diets.” → This statement is consistent with the discussion in the manuscript, but the lack on levels of malnutrition does not seem to be clearly connected to evidence in the current text. 
  - “ at the housing level it often results in diminished kitchen sizes and functionality. ”  → data could be provided.

 

- The following text could be improved for clarity:
  - “Another aspect of digitalization is linked to the provision of food information through technology, and the extent of information necessary for, and to be retained on, physical labels and information made available when purchasing foods online, and through meal delivery apps.” → I suggest to expand or rephrased for clarity.
  - Paragraph starting with “Multi-faceted policies and initiatives aimed at addressing urban poverty–including issues such as employment (…)” (p.11) → could benefit from some concrete examples on how the authors are thinking of these policies.

Does the paper provide a comprehensive overview of the policy and the context of its implementation in a way which is accessible to a general reader?

Yes

Is the discussion on the implications clearly and accurately presented and does it cite the current literature?

Yes

Are the recommendations made clear, balanced, and justified on the basis of the presented arguments?

Partly

Reviewer Expertise:

Systems thinking, food systems, sustainability, systems modeling.

I confirm that I have read this submission and believe that I have an appropriate level of expertise to confirm that it is of an acceptable scientific standard, however I have significant reservations, as outlined above.

# Reviewer report: Reviewer response for version 1

**DOI:** 10.5256/f1000research.179337.r391319

**Published:** 2025-08-23

**Authors:** Daniela Bernaschi

**Affiliations:** 1 University of Florence, Florence, Italy

**Summary of the Article**

 This policy-oriented perspective paper explores the complex interplay between urbanization, food systems, and public health. The authors argue that emerging and interrelated trends—housing development, digitalization, labour transitions, and dietary transitions—are reshaping urban food environments in ways that exacerbate health and nutrition inequalities. The paper advocates for holistic, city-led policy approaches that integrate public health goals into urban food systems. Its strength lies in combining policy relevance with a systems-thinking approach to urban health and nutrition.

**Major Comments**

**1. Section Structure and Titling**

 The current structure of the manuscript would benefit from clearer distinction between background framing and analytical content. The section titled *“Findings: Key Connections and Food Systems Trends Shaping Urban Health”* reads more as a conceptual overview than a findings section, particularly in the absence of empirical data. Renaming it to *“Background” *or *“Context”* and integrating it into the introduction could improve coherence. In contrast, the following section— *“Trends Shaping Urban Food Security and Nutrition”*—offers the strongest analytical contribution and might be more appropriately positioned as Section 1 of the main body.

**2. Sentence Length and Clarity**

 Certain parts of the manuscript, including boxed texts and explanatory paragraphs, contain overly long and complex sentences that affect readability. For example, in Box 1, the sentence preceding footnote 7 could be split into two or more parts for clarity. Streamlining these sections would improve the paper’s accessibility without compromising depth.

**3. Anchoring Trends with Data and References**

 Each of the four trends is articulated in a rich and conceptually grounded manner. However, their credibility would be significantly enhanced by including brief, concrete data points and specific references. This would help distinguish well-documented phenomena from more generalized interpretations and strengthen the evidence base of the paper.

**4. Methodological Transparency**

 Although the article is framed as a perspective piece, readers would benefit from a brief explanation of the literature review process. Outlining the scope—such as keyword selection, inclusion criteria, or timeframe—would add a layer of rigour and transparency. A short methodological note or paragraph would suffice.

**5. Positioning the “Holistic” Approach**

 The paper refers to existing urban food governance frameworks, including the Milan Urban Food Policy Pact (MUFPP) and the WHO Healthy Cities Initiative. Yet, the relationship between these initiatives and the proposed *“holistic city-led action”* remains underexplored. It would be helpful to clarify whether the authors are proposing a new model, an implementation strategy, or an integrative framework. Articulating how this approach builds on or departs from existing initiatives would enhance the originality and applicability of the contribution.

 6. **Clarify the Theoretical Grounding of “Holistic”**

 The term “holistic city-led action” is promising but remains somewhat vague. I encourage the authors to specify the theoretical underpinnings of this approach: Is it grounded in systems thinking, policy coherence, participatory governance, or a rights-based framework? Clarifying this would enhance conceptual precision and better situate the article within the literature on urban food policy and integrated local strategies, where holistic approaches are increasingly discussed in relation to food systems transformation, cross-sectoral coordination, and territorial inclusion

**7. Consider Power Relations in City-Level Implementation**

 While the paper emphasizes the importance of holistic, city-led action for transforming urban food systems, it could benefit from some reflection on the institutional and political limitations local governments face when pursuing systemic change. Even a brief mention of structural constraints—such as limited resources, fragmented mandates, or vertical power asymmetries—would offer a more nuanced picture of city-level capacity. This could help contextualize the potential and limits of municipal leadership in food system transformation.

**8. Acknowledge Critical Reflections in the Field**

 While the article offers a timely and policy-relevant contribution, a brief mention of ongoing debates within the field—particularly around the risks of technocratic approaches, fragmentation, or depoliticization in urban food policy—could strengthen its contextual grounding. Even a short acknowledgment of these tensions would help situate the proposal for holistic city-led action within the broader discursive landscape of urban food systems research. This addition is not essential, but could enrich the reader's understanding of the broader debates shaping urban food policies today.

**Minor Comments**

 -The abstract is informative and well written, but the four key structural trends (housing, digitalization, labour transitions, and dietary changes) only appear midway through. Introducing them earlier in the abstract could better signal the structure of the manuscript and highlight its analytical focus from the outset.

 -Given the number of interrelated trends and policy implications discussed, a visual element—such as a summary table or conceptual diagram—could help synthesize the information for readers. This would be especially valuable for policy audiences who may benefit from a more immediate overview of trends, impacts, and suggested interventions.

**Conclusion**

 This is a timely and thought-provoking contribution that highlights the evolving nexus between urbanization, food systems, and public health. By identifying four key structural transitions—housing, digitalization, labour, and diets—and linking them to urban food environments, the paper offers a valuable systems-thinking perspective on policy integration at the city level.

 Clarifying the structure of the manuscript, grounding each trend more clearly in data and literature, and elaborating the conceptual foundations of the proposed “holistic” approach would improve both its analytical clarity and its practical impact. A discussion of the political and institutional conditions shaping municipal action—along with a brief acknowledgment of ongoing debates in urban food policy—would further strengthen the manuscript’s relevance and reach.

 With these adjustments, the paper would make a meaningful contribution to current reflections on how cities can navigate complexity and lead food system transformations in ways that are context-sensitive, integrative, and equitable.

Does the paper provide a comprehensive overview of the policy and the context of its implementation in a way which is accessible to a general reader?

Yes

Is the discussion on the implications clearly and accurately presented and does it cite the current literature?

Partly

Are the recommendations made clear, balanced, and justified on the basis of the presented arguments?

Partly

Reviewer Expertise:

Food insecurity; Social inequalities; Measurement of food insecurity at the local level; Urban approaches to sustainable food systems; Environmentally and socially sustainable food systems.

I confirm that I have read this submission and believe that I have an appropriate level of expertise to confirm that it is of an acceptable scientific standard, however I have significant reservations, as outlined above.

# Reviewer report: Reviewer response for version 1

**DOI:** 10.5256/f1000research.179337.r393671

**Published:** 2025-07-26

**Authors:** Sandrine Simon

**Affiliations:** 1 University of the West of England, Bristol, UK

This is a very interesting article, presenting important recommendations for food and urban policies. The following notes give detailed feedback on the paper:

 The **title** perfectly reflects the objective of the paper: both in terms of how policies on food systems could act as *catalysts* for making cities more sustainable (‘paving the way’) – key message 9 does this very well too) and on highlighting the imperative to approach these issues from a holistic perspective.

 The fact that this article is included in the ‘Making the Case for urban health: defining value and relevance to contemporary challenges’ **collection** is very important: this article fits perfectly in this collection. This is also well mentioned and explained in the introduction.

**Abstract**

 “Rapid urbanisation is catalysing changes across the food system and, in turn, shaping urban diets” highlights very well the premise/ motivation for the research. The objectives are very well stated at the end of the abstract.

**Key messages:**

 point 2 needs to be reformulated/ punctuated differently: …through purchasing it, the food that is available and its relative price are key determinants of nutrition and health.

 I particularly appreciate point 4 (context specific) and the fact that the authors are not encouraging a ‘straight transferability’ of their approach and, instead, call for attention to be paid to specific needs emerged from specific contexts.

**Introduction**

 “…realization of several SDGs “: why not list them here?

 “…attainment of the Right to food” : yes, absolutely. Why not also mention *increased food security* (+ def. in footnote, as you did with the ‘Right to Food’) – especially because it appears in the heading of one of your sections (Trends in shaping urban food security and nutrition)?

 70% of food produced globally consumed by urban areas (ref.?)

 “While space to grow foods in urban areas is limited …:”: I think it would be worth here mentioning the dynamic body of research focusing on urban agriculture, including using roof tops, vertical farming, intensive sustainable agriculture, and urban regeneration projects that are working on optimising and increasing urban space that could be used for growing food and making cities less food dependant.

 Feed Vs *nourish *very much appreciated here. Nice touch. Would it be worth mentioning a *‘regenerative’* objective as a (light) way to signal connections with research on regenerative urban planning or regenerative agriculture?

 At the end of the introduction, I feel it would be useful to indicate *which structure* your paper will adopt to articulate your thoughts/ narrative.

 Having printed the paper in black and white, I think (although I am not 100% sure) that

 Part 1: I. Findings: key connections and food systems trends shaping urban health over the coming decade (with only one sub section? I.1. Food systems and urban health key connections). Why put this as a sub-section if there is only one?

 Part 2: II. Trends shaping urban food security and nutrition

 With sub-sections as follows: II.1. Housing development; II.2. Digitalization; II.3. Labour transitions, particularly in the food sector ; II.4. Dietary transitions

 Part 3 III. Key challenges and future opportunities for urban food systems policies

 With a long initial section, followed by what would then be untitled III.1. How can a holistic approach to urban food system address health and social challenges? (I feel that the section before this needs a title)

 Part 4 IV. Knowledge gaps and research needs (“ *Knowledge gaps to address in future research*”?) I am suggesting this alternative title for this last section as one normally sees ‘knowledge gaps’ being mentioned at the beginning of an article to demonstrate how research questions were developed to carry out the research being talked about in this paper.

 Intuitively and before reading the whole paper, I wonder why Part II does not come before Part I to highlight the problems to address. Then Part 1 (Finding key connections) would start investigating potential ways of addressing the problem?

 Similarly, I wonder whether Part 4 and conclusions could be merged?

**Part 1.**

 I would change the title of this part to: “Food systems and urban health: key connections and trends shaping urban health over the coming decade”. No sub-title needed, only one main section for this part.

 2d parag. “The greatest levels of urban food insecurity…respectively), while it affects 11.8% of Northern Americans and 8.1% of Europeans.

 4 ^th^ parag. Social and environmental structures : social and environmental factors – maybe better?

 Last paragraph (as a way of introducing next part), last sentence “As urban populations grow…..impact on health as well as their trend in order to develop effective strategies for addressing these pressing issues”.

**Part 2**

 1 ^st^ sentence: change to “Various studies have explored the diverse environmental……..  food systems and their associated health outcomes ….above (ref.8 & 15 – maybe add one or two more)”. 2 ^nd^ sentence “… that, to date, have not been well   “ (add comas)

 Absolutely impossible to read Figure 1 – could you please make it a bit bigger?

 Section II.2 (Digitalization) : 4 ^th^ paragraph: why is ref 4 [4] presented differently from the other refs.?

 Last paragraph: I would add ‘Finally, another aspect of ..

 Section II.3

 2d paragraph: widely criticized (not critiqued)

 More data would help illustrate your points in Part 2.

**Part 3**: Here it would make sense to have two sub-parts.

 III. Key challenges and potential opportunities for urban food systems policies

 III.1. implications and challenges for urban policies

 III.1.1 Urban power structures

 III.1.2. The need for urban policy coherence

 III.1.3. Enacting transitions

 III.2. How can a holistic integrated approach to urban food system address health and societal challenges?

 Maybe give **more insights on the lessons learnt from the WHO’s health in all policies frame**work and how this could followed in the more specific context of food policies?

 I feel that **diagrammatic representations (systems map maybe**?) could help to illustrate the interrelations you are talking about and summarise the main points of the paper.

**Part 4 to combine with the conclusion**?

 In the **conclusion**, you mention the fact that ‘City governments’ involvement in food policy has thus blossomed, with city serving as places for experimentation and innovation, …’: You could have presented some of these apparently existing examples and these types of new governance processes and initiatives that address the issues you discuss in this paper could have been critically explored with suggested recommendations? They could have served as illustrative examples?

Does the paper provide a comprehensive overview of the policy and the context of its implementation in a way which is accessible to a general reader?

Yes

Is the discussion on the implications clearly and accurately presented and does it cite the current literature?

Yes

Are the recommendations made clear, balanced, and justified on the basis of the presented arguments?

Partly

Reviewer Expertise:

urban agriculture; ecological economics; environemntal governance

I confirm that I have read this submission and believe that I have an appropriate level of expertise to confirm that it is of an acceptable scientific standard, however I have significant reservations, as outlined above.

# Reviewer report: Reviewer response for version 1

**DOI:** 10.5256/f1000research.179337.r393669

**Published:** 2025-06-28

**Authors:** Chavula Petros

**Affiliations:** 1 Haramaya University, Dire Dawa, Ethiopia

1. Abstract (p. 1, ¶ 2)

- Comment: The abstract succinctly outlines the problem and proposed interventions but omits explicit mention of the four emerging trends.
- Recommendation: On p. 1, ¶ 2, insert a brief clause naming “housing transformations, digitalization of food access, labour shifts in the gig economy, and dietary transitions” to guide readers from the outset.

2. Introduction (p. 2, ¶ 1–3)

- Relevance & Framing: The opening paragraphs (p. 2, ¶ 1) effectively set the context of rapid urbanization and health inequities. However, p. 2, ¶ 2 repeats similar statistics on urban food insecurity.
- Recommendation: Consolidate p. 2, ¶ 2–3 into a single paragraph, citing FAO (2022) only once, to improve flow and reduce redundancy.

3. Study Methods (p. 4, ¶ 1)

- Current State: As a perspective, the manuscript lacks a formal methods section.
- Recommendation: Add a “Methods” subsection on p. 4, ¶ 1, detailing your literature search strategy—databases searched, keywords, date range, and inclusion criteria—to enhance transparency and reproducibility.

4. Relevance & Academic Importance (p. 3, ¶ 4–5)

- Strength: The comparison with existing frameworks is implied but not explicit.
- Recommendation: In p. 3, ¶ 5, insert a sentence contrasting this framework with the Milan Urban Food Policy Pact (2015) to clarify how your “holistic city-led action” extends prior work.

5. Trends Analysis (pp. 5–7)

- Depth: Each trend is well described, but lacks quantitative anchors.
  - Housing (p. 5, ¶ 2): Reference percent reduction in kitchen size in London high-density blocks (e.g., “–12 m² average”) with citation.
  - Digitalization (p. 6, ¶ 1): Cite McKinsey’s report on 45 % growth in online grocery orders.
  - Recommendation: Add one data point and citation per trend at the start of each corresponding paragraph (pp. 5–7).

6. References & Citations (pp. 12–15)

- Issue: Several entries repeat the same Crossref URL across multiple citations (e.g., pp. 13–14).
- Recommendation: Consolidate duplicates into single reference entries, ensure every citation includes a DOI, and replace at least two grey-literature sources with peer-reviewed articles (e.g., substitute a McKinsey statistic with a Journal of Urban Health study).

7. Grammar & Style (p. 2, ¶ 4; p. 8, ¶ 3)

- Examples:

“Urban areas are also often sources of social determinants of poor health with inequalities driven by individual and household-level factors…” (p. 2, ¶ 4)

- Recommendation: Split this into two sentences. Also, correct the typo “Themanifestations” on p. 8, ¶ 3 to “The manifestations.”

8. Structure & Flow (throughout)

- Observation: The flow from trends to solutions is logical but would benefit from a summary table.
- Recommendation: After p. 9, ¶ 2, insert Table 1 summarizing each recommendation, its responsible governance level, and key metrics for monitoring.

9. Constructive Tone & Objectivity (throughout)

- Commendation: The manuscript maintains respectful language.
- Recommendation: When discussing private-sector roles (p. 7, ¶ 2), balance critiques with successful examples (e.g., Copenhagen’s healthy school meal policy, p. 7, ¶ 3).

10. Conclusion (p. 10, ¶ 1)

- Strength: The call to action is clear.
- Recommendation: In p. 10, ¶ 1, briefly restate the four trends and the need for integrated city governance to reinforce the main message.

Overall

 This manuscript offers a timely, policy-oriented synthesis. Incorporating the above page- and paragraph-specific revisions—especially enhancing methodological transparency (p. 4, ¶ 1), tightening prose (p. 2, ¶ 2–3), and refining citations (pp. 12–15)—will strengthen its academic rigor and practical utility.

Does the paper provide a comprehensive overview of the policy and the context of its implementation in a way which is accessible to a general reader?

Yes

Is the discussion on the implications clearly and accurately presented and does it cite the current literature?

Yes

Are the recommendations made clear, balanced, and justified on the basis of the presented arguments?

Yes

Reviewer Expertise:

Climate Change, Climate-Smart Agriculture and Agricultural Economics

I confirm that I have read this submission and believe that I have an appropriate level of expertise to confirm that it is of an acceptable scientific standard.
